# A Universal Detection Method for Adversarial Examples and Fake Images

**DOI:** 10.3390/s22093445

**Published:** 2022-04-30

**Authors:** Jiewei Lai, Yantong Huo, Ruitao Hou, Xianmin Wang

**Affiliations:** 1 School of Computer Science and Cyber Engineering, Guangzhou University, Guangzhou 510006, China; 1906200093@e.gzhu.edu.cn (J.L.); 1906300038@e.gzhu.edu.cn (Y.H.); 2 Pazhou Lab, Guangzhou 510330, China; 3 Institute of Artificial Intelligence and Blockchain, Guangzhou University, Guangzhou 510006, China; xianmin@gzhu.edu.cn

**Keywords:** adversarial example, deep forgery, detection

## Abstract

Deep-learning technologies have shown impressive performance on many tasks in recent years. However, there are multiple serious security risks when using deep-learning technologies. For examples, state-of-the-art deep-learning technologies are vulnerable to adversarial examples that make the model’s predictions wrong due to some specific subtle perturbation, and these technologies can be abused for the tampering with and forgery of multimedia, i.e., deep forgery. In this paper, we propose a universal detection framework for adversarial examples and fake images. We observe some differences in the distribution of model outputs for normal and adversarial examples (fake images) and train the detector to learn the differences. We perform extensive experiments on the CIFAR10 and CIFAR100 datasets. Experimental results show that the proposed framework has good feasibility and effectiveness in detecting adversarial examples or fake images. Moreover, the proposed framework has good generalizability for the different datasets and model structures.

## 1. Introduction

In recent years, as one of the core technologies of artificial intelligence, deep learning has attracted unprecedented attention from academia and industry [1]. Compared with traditional machine learning methods, deep learning produces results with higher accuracy, does not require complex feature engineering, and has better adaptability. Hence, deep-learning technology has been gradually applied to various fields, such as computer vision, speech recognition, natural language processing, autonomous driving, etc., [2,3,4,5]. However, research shows that deep learning still has many problems in its security and privacy [6,7,8,9], such as adversarial examples and deep forgery [10,11].

Szegedy et al. first proposed the concept of adversarial examples [12]. Its basic principle is to add some specific subtle perturbations to the original data; the model would output error results with high confidence. The discovery of adversarial examples illustrates the fragility of deep-learning models. Since then, the researchers have researched adversarial examples and proposed many adversarial example generation methods, such as FGSM, C&W, DeepFool, etc., [13,14,15,16]. These methods can generate adversarial examples with extremely high success rates based on different attack scenarios and targets. Moreover, it was found that the adversarial examples are transferable, i.e., the adversarial examples generated for one model are effective for other similar models [17]. This aggravates the seriousness of deep learning security problems and greatly restricts the application of deep-learning technology in military, medical, financial, and other sophisticated fields [18,19,20].

Except for the security issues of the technology itself, deep learning has abuse problems, such as deep forgery. Deep forgery uses deep-learning algorithms [11], i.e., generative adversarial networks (GANs), to tamper with or forge original data so that observers mistakenly regard fake data as original data. These fake data are realistic, diversified, and challenging to identify. With the help of the ripple effect of online social media, fake data is likely to spread on a large scale, causing a severe social impact. More seriously, if criminals use deep forgery for political or malicious profit-making motives, it will result in many risks and severe threats to political, economic, social, and national security. Hence, the detection of adversarial examples and fake images is a hot issue in academia and industry.

To solve the above issue, we explore the detection methods for adversarial examples and fake images. Inspired by membership inference attacks that use the model output to determine whether a sample belongs to the training dataset [21], we observe that there are also differences between the model outputs of normal samples and adversarial examples (fake images). Specifically, the distribution of model outputs is fit using the kernel density estimation. We propose a universal detection method for adversarial examples and fake images based on this difference. This method includes the detector training algorithm and online detection algorithm. The detector training algorithm is used to construct the model output’s dataset and train the detector. This dataset consists of the model outputs of normal samples and adversarial examples (fake images). In addition, the detector is trained to learn the rule of the output distribution of normal samples and adversarial examples (fake images). The online detection algorithm is to obtain the model outputs of samples and calculate detection results. To the best of our knowledge, Li’s method is similar to ours [22]. However, he uses the middle-layer features of the Bayesian neural network to determine normal samples and adversarial examples, and we use the output of the deep neural network. Moreover, our method is not only suitable for the detection of adversarial examples, but also for the detection of fake images.

Our contributions consist of the following:

1. Based on the difference in the distribution of model outputs between normal samples and adversarial examples (fake images), we propose a universal detection method for adversarial examples and fake images.
2. We tested the detector’s performance using state-of-the-art generation algorithms of adversarial examples and fake images and proved the effectiveness of the detector.
3. We tested the proposed method on different datasets and neural network structures and proved the generalizability of the detector.

The rest of this paper is structured as follows. Section 2 introduces related research. Section 3 presents our method. Section 4 experimentally evaluates our method. Section 5 summarizes our work.

## 2. Related Work

In this section, we introduce the related work of adversarial examples and fake images from attack and defense.

### 2.1. Adversarial Examples

#### 2.1.1. Attack

The generation methods of adversarial examples can be roughly divided into two types: gradient-based methods and optimization-based methods.

Gradient-based methods.The basic principle of this type of method is to add perturbations in the gradient direction, and these perturbations should not be easily noticed. FGSM is a one-step method that adds limited perturbations in the gradient direction to search for a similar image, which will cause the model to output wrong results [13]. BIM, also called I-FGSM, which is a multiple-iterations version of FGSM, extends FGSM by running a minor optimization in each iteration [23]. PGD is also an iteration extension of FGSM [24]. Different from BIM directly clipping pixel values, it first utilizes gradient projection to avoid excessive changes in the optimization process.

Optimization-based methods. Different from gradient-based methods, its basic principle is to take the generation process of adversarial examples as a constrained optimization problem, that is, to ensure that the model outputs wrong results without being easily noticed. DeepFool adds some minor perturbation to the normal image, causing the image to exceed the classification boundary through iterative calculation [15]. Similar to DeepFool, UAP also uses adversarial perturbations to make normal images exceed the classification boundary [25]. It can be added into most images to generate adversarial examples and have good generalization capabilities on other network architectures.

#### 2.1.2. Defense

The defense methods against adversarial examples mainly adopt two strategies: robust classifier and detector-based methods.

Robust classifier methods. The robust classifier method improves the robustness of the model, such as defensive distillation [14] and adversarial training [13]. For defensive distillation, the model can be smoother than the original model by assigning a larger value of *T* during the student model training stage, which would reduce the sensitivity to perturbations and improve the robustness and generalizability of the model. Adversarial training is currently the most effective defense method. Its basic idea is to assign the correct labels to adversarial examples and then train the model with these samples to improve its robustness. It has also been proved that adversarial training can provide higher precision and regularization for models [13]. However, adversarial training also presents some challenges. Adversarial training needs to generate many adversarial examples, so the computational cost and time cost of the method are very high. The adversarial example generation methods are constantly updated, which leads to the continuous retraining of the model.

Detector-based methods. This strategy trains a detector to distinguish between adversarial examples and normal samples. Feinman et al. believe that the adversarial examples deviate from the manifold region of the real data, so they use the kernel density estimation function in the feature space of the middle layer to detect abnormal points that deviate from the data manifold [26]. Ma et al. observed that the Local Intrinsic Dimension of the hidden layer output differs between normal images and adversarial examples. They used this characteristic to detect adversarial examples [27]. Tian et al. found that image processing operations could invalidate the adversarial examples, which would not affect the classification of the normal images [28]. MagNet constructs multiple autoencoders and uses the reconstruction errors of autoencoders to detect adversarial examples based on cryptographic randomness [29]. SafetyNet detects adversarial examples that rely on neural activation patterns with SVM [30]. Li et al. found that the output distribution of the hidden layer of the adversarial examples was different from that of the normal images, so they used the Bayesian neural network to simulate the output distribution of the hidden layer and detect the adversarial examples using distribution dispersion [22].

### 2.2. Fake Images

#### 2.2.1. Attack

Traditional image forgery methods are generated mainly by image-editing software. With the development of GANs, GANs-based generation methods for fake images have become the mainstream.

Software-based method. It is straightforward to use powerful image editing software to generate fake images without leaving perceptible artifacts. Those methods can be divided into the copy-move and splice methods, which create fake images without leaving traces by adding new content to the original image or perform image stitching, respectively. In addition, the copy-move method is the mainstream method that can change the entire meaning of the original image.

GANs-based method. GANs is a method of unsupervised learning that Goodfellow proposes. It consists of a generator and a discriminator. Taking the normal image or the generator’s output as input, the discriminator distinguishes the generator’s output from the real sample as much as possible. The generator randomly samples from the latent space as input and tries to imitate the real samples in the training set to fool the discriminator. Two neural networks learn by confronting each other and constantly adjusting the parameters. With the development of GANs technology, GANs are used for different scenarios, for example, generating high perceptual quality images, domain transfer, image to image translations, and so on [31].

#### 2.2.2. Defense

Defense methods against fake images are roughly divided into defense against software-based fake images and defense against GANs-based fake images.

Defense against the software-based fake image. The methods of image-manipulation detection can be summarized into two types: (i) Active: people embed some additional information, i.e., digital watermarks or digital fingerprinting, into the image to determine the authenticity of the image [32]. However, active approaches have some shortcomings, such as difficulty in secondary propagation and single verification, which are confronted with overlooked challenges. (ii) Passive: passive approaches extract the features from the images and use these features for forgery detection [33]. For example, it can identify contrast enhancement, reveal image resampling, etc. To solve the problem that an image may use multiple tampering methods, the authors in [34] use various features in a steganalysis to detect the fake image and identify tampering types. However, these traditional approaches are mostly ineffective when identifying GANs-based fake images.

Defense against GANs-based fake image. Similar to adversarial-example detection, the most direct method of detecting fake images is to train the detector using real and fake data. Marra et al. show that a simple fake image detector could be constructed using an image translation network [35]. A three-channel co-occurrence matrix-based detection method was proposed in [36]. Dang et al. realized that the detection of fake face images and the location of the tampered region depend on an attention mechanism [37]. Some researchers believe that, due to the diversity of fake images and the continuous updating of generation methods, the detector could only distinguish the fake images in training. To enhance the generalizability of the detector, Zhang et al. proposed a fake image-generation method called AutoGAN [38]. This method uses a frequency-spectrum input instead of pixel-space input to train the detection model. On the contrary, Wang et al. found that only using a fake image and then performing data pre-processing or data enhancement to expand the training data set could improve the generalizability of the detector [39].

## 3. Method

In this paper we mainly focus on the detection of adversarial examples and GANs-based fake images, and we present our method in detail in this section.

### 3.1. Overview

#### 3.1.1. Observation

**Figure 1:**
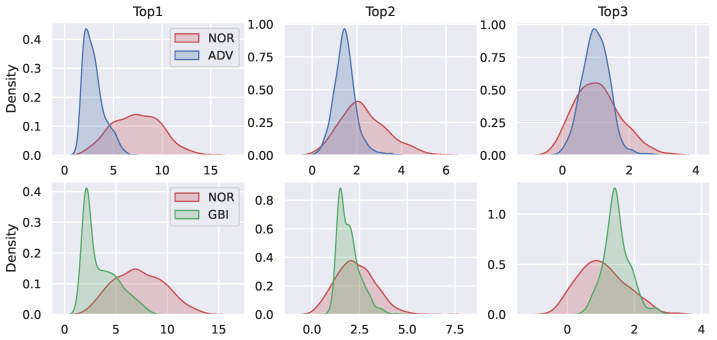
The difference of the distribution of model outputs between normal samples and adversarial examples (fake images).

We observed the difference in the distribution of model outputs between adversarial examples (fake images) and normal samples through experiments. Specifically, we first obtained the output of Googlenet on normal samples, adversarial examples, and fake images, then used kernel density estimation to fit the distribution of model outputs. The result is shown in the Figure 1.

In Figure 1, TOP1, TOP2, and TOP3 indicate the top 1 value, the top 2 values, and the top 3 values of the model outputs, respectively. NOR, ADV, and GBI represent normal data, adversarial examples, and fake images, respectively. ADV and GBI are generated by DeepFool [15] and WGAN [40], respectively. It can be seen from Figure 1 that there are significant differences in the distribution of model outputs between the normal images and the adversarial examples (fake images).

#### 3.1.2. Framework

**Figure 2:**
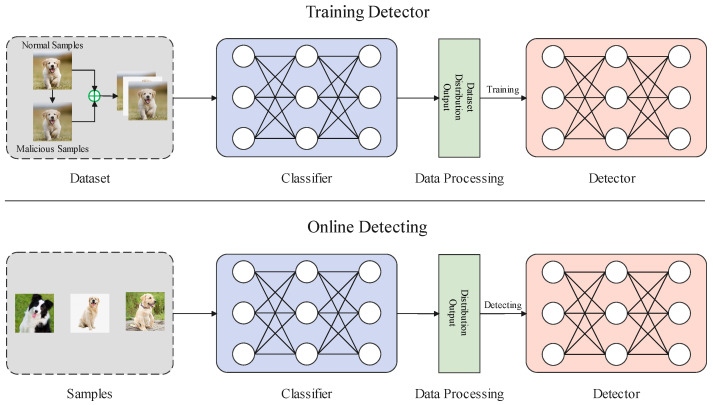
The framework of our method.

Based on the above observation, we propose a universal detection method for adversarial examples and fake images. The overall framework is shown in Figure 2.

Figure 2 shows that the method mainly includes two stages: detector training and online detection. The purpose of detector training is to learn the difference in the distribution of model outputs between the normal samples and adversarial examples (fake images) to distinguish the normal samples from adversarial examples or fake images. Therefore, we need to generate a certain number of adversarial examples or fake images in advance as training data. Moreover, to reduce the time-cost of training and improve the detection efficiency, we chose the first *k* values from the model output to represent the distribution of outputs. The purpose of online detection is to detect adversarial examples or fake images. The following is a detailed introduction to detector training and online detection.

### 3.2. Detector Training

The main work of detector training includes constructing the training dataset and training detector, and the basic process is shown in Algorithm 1.
**Algorithm 1** Detector Training Algorithm.**Require:** 
Normal Data $D_{n}$, Generator G, Target Model F  1:$D_{m}\leftarrow G\left(D_{n}\right)$  2:D← Merge$\left(D_{m},D_{n}\right)$  3:$D_{out}\leftarrow F\left(D\right)$  4:$D_{train}\leftarrow$ Top$\left(k,D_{out}\right)$  5:D← Train$\left(D,D_{train}\right)$

According to Algorithm 1, normal data, the malicious data generator, and the target model, F, should be obtained before training the detector. Specifically, we first use G to generate adversarial examples or fake images, as shown in Line 1. G represents some mainstream adversarial examples or fake-image-generation methods. Line 2 merges normal data and adversarial examples (fake images). Line 3 inputs the merged data *D* into F to obtain the output distribution dataset $D_{out}$. In our experiments, F is the classifier trained on the CIFAR10 or CIFAR100 datasets. To reduce the complexity of the detector and improve the detection efficiency, we selected the top *k* values from the model output to represent the output distribution, as shown in Line 4. Line 5 uses the dataset $D_{train}$ to train the detector D. The detector can be trained offline and deployed online to reduce the time-cost.

### 3.3. Online Detection

The main work of online detection includes obtaining the model output of the untrusted data and computing the detection result. The basic process is shown in Algorithm 2.
**Algorithm 2** Online Detection Algorithm.**Require:** 
Untrusted Data $D_{u}$, Target Model F, Detector D  1:$D_{uout}\leftarrow F\left(D_{u}\right)$  2:$D_{uk}\leftarrow$ Top$\left(k,D_{uout}\right)$  3:$Result\leftarrow D(D_{uk})$

According to Algorithm 2, Line 1 obtains the model output of untrusted data $D_{u}$. Line 2 selects the top *k* values from $D_{uout}$ to obtain $D_{uk}$. Finally, $D_{uk}$ is input into the detector D to obtain the detection result.

## 4. Experiments

In this section, we experimentally evaluate the proposed method using the CIFAR10 and CIFAR100 datasets. Both CIFAR10 and CIFAR100 are natural image datasets, and both include 50,000 training images and 10,000 test images. However, there are ten classes in CIFAR10 and 100 classes in CIFAR100. The experiments include performance experiments, generalizability experiments, and transferability experiments. Additionally, we use AUC as a measure of our detector’s performance in our experiments. Next, we will introduce the experiments in detail.

### 4.1. Performance Experiments

The performance experiments were used to test the ability of our method in adversarial example detection or fake image detection.

#### 4.1.1. Performance in Adversarial Example Detection

We used nine state-of-the-art adversarial example-generation methods for detection, including FGSM, DeepFool, BIM, PGD, C&W, etc., [13,14,15,23,24]. The experimental results are shown in Table 1 and we mark out the best detection result using bold text for each type of adversarial example.

From Table 1, we know: (1) Given the generation methods of adversarial examples in training, if these methods are the same as the generation methods in the evaluation, the detector almost reached the highest level of detection accuracy; on the contrary, the detection accuracy decreased slightly. (2) On the whole, when the generation method in training is ZOO or NewtonFool, the detector reaches the best detection performance. Hence, in the real world, we could use ZOO or NewtonFool to generate adversarial examples in training.

#### 4.1.2. Performance in Fake Image Detection

Considering that the mainstream generation methods of fake images are mainly based on GAN, we selected eight state-of-the-art GAN-based algorithms to test the detector’s performance. The experimental results are shown in Table 2 and we mark out the best detection result using bold text for each type of fake image.

It can be seen from Table 2 that the detection results of fake images are similar to the adversarial examples. Given the generation methods of fake images in training, if these methods are the same as the generation methods in the evaluation, the detector achieved the best detection performance; on the contrary, the detection accuracy decreased slightly. In addition, when the generation method in training was WGAN_DIV [40], the detector reached the best detection performance. Hence, WGAN_DIV is a candidate to generate fake images in training in the real world.

### 4.2. Generalizability Experiments

The generalizability of the detector mainly includes (1) the generalizability of datasets; (2) the generalizability of target model architecture. In addition, the generation methods of adversarial examples and fake images are DeepFool and WGAN [15,40], respectively. In addition, we have to describe the experiment case in the form of condition I–condition II. For example, CIFAR10–CIFAR100 represents that the detector is trained by CIFAR10 and evaluated by CIFAR100. Limited by the two time-scale update rule (TTUR), we only use AutoGAN [41] and TransGAN [42] generators on the CIFAR10 dataset. Below, we provide a detailed introduction to the experimental content.

#### 4.2.1. Generalizability for Dataset

**Figure 3:**
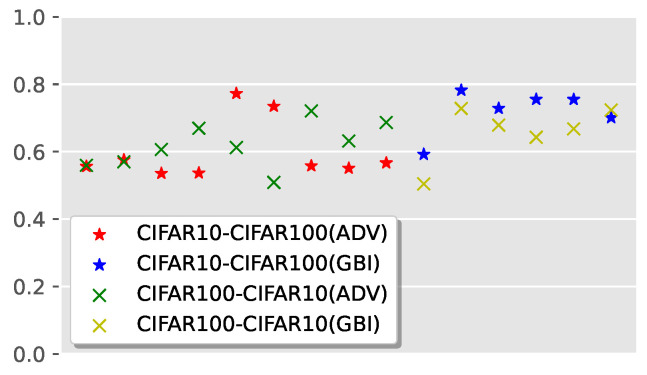
The generalizability for dataset.

We alternately used CIFAR10 and CIFAR100 as the training dataset and used CIFAR100 and CIFAR10 as the evaluation dataset to verify the generalizability of the detector for the dataset. The experimental results are shown in Figure 3.

As shown in Figure 3, the AUC values of adversarial example detection are mainly distributed between 0.5 and 0.8; most of the AUC values of fake image detection are between 0.6 and 0.8. Hence, the generalizability of the fake image detector is slightly stronger than that of the adversarial example detector, but it still needs to be further improved.

#### 4.2.2. Generalizability for Target-Model Architecture

**Figure 4:**
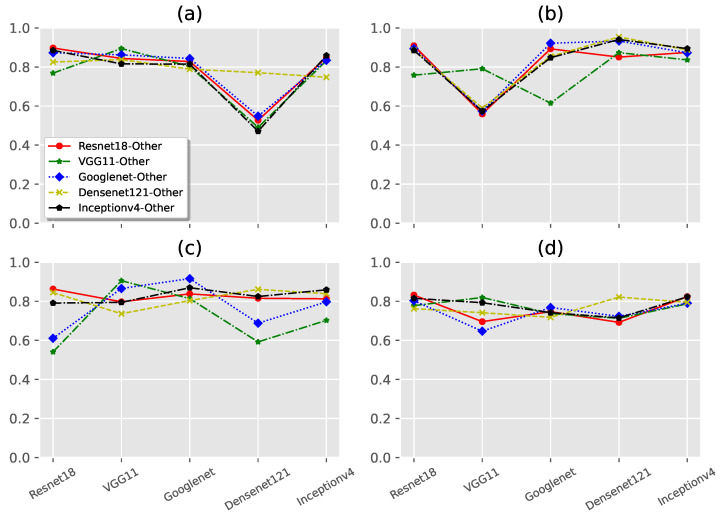
The generalizability for target model architecture. (**a**,**b**) are the experimental results of ADV on CIFAR10 and CIFAR100. (**c**,**d**) are the experimental results of GBI on CIFAR10 and CIFAR100.

We selected five neural network structures to verify the generalizability of the target model structure, including Resnet18, VGG11, Googlenet, Densenet121, and Inceptionv4. Specifically, we first selected one of the network structures as the target model, one after the other in turn, then used the output of the target model to train the detector, and finally used the remaining networks to evaluate the generalizability of the detector. The experimental results are shown in Figure 4.

From Figure 4, the adversarial example detector shows good generalizability for different model structures. However, there are a few cases below the average level. For example, the generalizability for Densenet121 on the CIFAR10 dataset, the generalizability for VGG11 and Googlenet on the CIFAR100 dataset, etc. Similar to the adversarial example detector, the generalizability of the fake image detector for the model structure is generally reasonable.

### 4.3. Transferability Experiments

**Figure 5:**
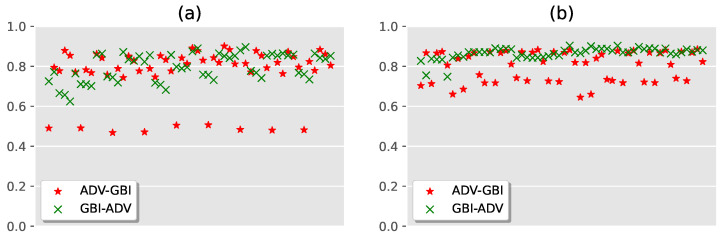
The result of transferability experiments. (**a**) is the experimental result on CIFAR10. (**b**) is the experimental result on CIFAR100.

The transferability experiments include (1) testing the ability of the adversarial example detector to detect fake images; (2) testing the ability of the fake image detector on adversarial examples. The experimental results are shown in Figure 5.

As shown in Figure 5, for the CIFAR10 dataset, the AUC values of the detectors are mainly distributed from 0.7 to 0.9, but the fake image detector is more stable. For the CIFAR100 dataset, the transferability of the fake image detectors is significantly better than that of adversarial example detectors, all distributed around 0.9. This shows that our method has good transferability, and a single detector can be used to simultaneously detect adversarial examples and fake images to a certain extent.

## 5. Conclusions

In this paper, we observe the difference in output distribution between normal samples and adversarial examples (fake images) and propose a universal detection method for adversarial examples and fake images. The method mainly includes two stages: detector training and online detection. In the detector-training stage, we used the output distribution of normal and adversarial samples (fake images) to train the adversarial example (fake image) detectors. After training the detector, we took the model output of untrusted data as the input of the detector to realize online detection of adversarial examples or fake images. We experimentally verified our method using CIFAR10 and CIFAR100 datasets, and the results show that: (1) the detector has a good detection ability for adversarial or fake images; (2) the detector has good generalizability for different model structures; and (3) the detector has good transferability, that is, the adversarial example detector and fake images detector can effectively detect fake images or adversarial examples. Hence, in the real world, our method is feasible and effective.

## Figures and Tables

**Table 1:** The results of adversarial example detection (The vertical axis and the horizontal axis represent training and evaluation, respectively).

CIFAR10									
	FGSM	DeepFool	BIM	PGD	AutoPGD	UPA	NewtonFool	ZOO	C&W
FGSM	**0.905**	0.911	0.796	0.781	0.757	0.817	0.873	0.869	0.875
DeepFool	0.898	**0.916**	0.799	0.784	0.756	0.796	0.875	0.866	0.871
BIM	0.884	0.887	**0.833**	0.811	0.776	0.802	0.871	0.865	0.872
PGD	0.887	0.890	0.832	**0.813**	0.813	0.780	0.804	0.876	0.865
AutoPGD	0.867	0.877	0.778	0.769	0.783	0.871	0.853	0.846	0.862
UPA	0.860	0.872	0.744	0.715	**0.880**	**0.880**	0.835	0.826	0.837
NewtonFool	0.903	0.907	0.817	0.803	0.774	0.829	**0.889**	0.877	0.895
ZOO	0.902	0.906	0.820	0.803	0.774	0.827	0.888	**0.879**	0.892
C&W	0.901	0.905	0.817	0.800	0.772	0.841	0.888	0.876	**0.896**
**CIFAR100**									
	FGSM	DeepFool	BIM	PGD	AutoPGD	UPA	NewtonFool	ZOO	C&W
FGSM	**0.882**	0.910	0.869	0.864	0.875	0.907	0.880	0.888	0.883
DeepFool	0.871	**0.922**	0.856	0.855	0.854	0.910	0.874	0.870	0.871
BIM	0.879	0.913	**0.874**	0.864	0.877	0.906	0.889	0.891	0.887
PGD	0.877	0.903	0.871	**0.873**	0.876	0.894	0.885	0.890	0.890
AutoPGD	0.878	0.908	0.869	0.864	**0.880**	0.902	0.888	0.888	0.887
UPA	0.865	0.865	0.843	0.842	0.848	**0.917**	0.855	0.864	0.859
NewtonFool	0.873	0.904	0.869	0.867	0.867	0.891	**0.889**	0.889	0.887
ZOO	0.880	0.909	0.870	0.870	0.877	0.902	0.885	**0.894**	0.887
C&W	0.879	0.879	0.873	0.871	0.877	0.895	0.886	0.891	**0.892**

**Table 2:** The results of fake image detection (The vertical axis and the horizontal axis represent training and evaluation, respectively).

CIFAR10								
	GAN	ACGAN	WGAN	WGAN_GP	WGAN_DIV	DCGAN	AutoGAN	TransGAN
GAN	**0.743**	0.680	0.776	0.789	0.783	0.680	0.579	0.518
ACGAN	0.495	**0.855**	0.798	0.892	0.874	0.827	0.624	0.562
WGAN	0.548	0.775	**0.830**	0.885	0.875	0.758	0.602	0.555
WGAN_GP	0.505	0.806	0.808	**0.906**	0.886	0.786	0.604	0.545
WGAN_DIV	0.512	0.787	0.811	0.900	**0.891**	0.780	0.599	0.535
DCGAN	0.502	0.844	0.795	0.878	0.868	**0.839**	0.637	0.554
AutoGAN	0.551	0.787	0.778	0.813	0.796	0.785	**0.647**	0.585
TransGAN	0.579	0.765	0.761	0.789	0.764	0.754	0.638	**0.586**
**CIFAR100**								
	GAN		ACGAN	WGAN	WGAN_GP	WGAN_DIV		DCGAN
GAN	**0.822**		0.839	0.740	0.827	0.842		0.825
ACGAN	0.716		**0.877**	0.711	0.866	0.877		0.809
WGAN	0.786		0.837	**0.768**	0.845	0.853		0.818
WGAN_GP	0.734		0.869	0.726	**0.871**	0.879		0.822
WGAN_DIV	0.739		0.872	0.726	0.868	**0.885**		0.824
DCGAN	0.762		0.863	0.747	0.866	0.877		**0.847**

## Data Availability

Not applicable.
